# Persistent hypoxia promotes myofibroblast differentiation via GPR‐81 and differential regulation of LDH isoenzymes in normal and idiopathic pulmonary fibrosis fibroblasts

**DOI:** 10.14814/phy2.15759

**Published:** 2023-08-31

**Authors:** Richard S. Nho, Cami Rice, Jayendra Prasad, Hannah Bone, Laszlo Farkas, Mauricio Rojas, Jeffrey C. Horowitz

**Affiliations:** ^1^ Division of Pulmonary, Critical Care and Sleep Medicine, Department of Internal Medicine, The Davis Heart and Lung Research Institute The Ohio State University Columbus Ohio USA

**Keywords:** G protein‐coupled receptor‐81 (GPR‐81), hypoxia, idiopathic pulmonary fibrosis (IPF), lactate dehydrogenase, α‐smooth muscle actin (α‐SMA)

## Abstract

Hypoxia, a state of insufficient oxygen availability, promotes cellular lactate production. Lactate levels are increased in lungs from patients with idiopathic pulmonary fibrosis (IPF), a disease characterized by excessive scar formation, and lactate is implicated in the pathobiology of lung fibrosis. However, the mechanisms underlying the effects of hypoxia and lactate on fibroblast phenotype are poorly understood. We exposed normal and IPF lung fibroblasts to persistent hypoxia and found that increased lactate generation by IPF fibroblasts was driven by the FoxM1‐dependent increase of lactate dehydrogenase A (LDHA) coupled with decreased LDHB that was not observed in normal lung fibroblasts. Importantly, hypoxia reduced α‐smooth muscle actin (α‐SMA) expression in normal fibroblasts but had no significant impact on this marker of differentiation in IPF fibroblasts. Treatment of control and IPF fibroblasts with TGF‐β under hypoxic conditions did not significantly change LDHA or LDHB expression. Surprisingly, lactate directly induced the differentiation of normal, but not IPF fibroblasts under hypoxic conditions. Moreover, while expression of GPR‐81, a G‐protein‐coupled receptor that binds extracellular lactate, was increased by hypoxia in both normal and IPF fibroblasts, its inhibition or silencing only suppressed lactate‐mediated differentiation in normal fibroblasts. These studies show that hypoxia differentially affects normal and fibrotic fibroblasts, promoting increased lactate generation by IPF fibroblasts through regulation of the LDHA/LDHB ratio and promoting normal lung fibroblast responsiveness to lactate through GPR‐81. This supports a novel paradigm in which lactate may serve as a paracrine intercellular signal in oxygen‐deficient microenvironments.

## 1 INTRODUCTION

Mitochondrial dysfunction and aberrant cellular metabolism have emerged as critical drivers of many disease processes in humans, including cancer, diabetes, and fibrosis of the lungs and other organs (Alcalá et al., 2017; Loomba et al., 2021; Watanabe et al., 2008). Under homeostatic conditions, cellular energy requirements are largely met by mitochondrial respiration driven by the tricarboxylic acid (TCA) cycle. Glucose is imported into cells and converted to pyruvate in the cytoplasm via glycolysis. Pyruvate is then transported into mitochondria and converted to Acetyl‐CoA which enters the TCA cycle and serves as the substrate for multiple metabolic intermediates and the generation of NADH and FADH_2_. These molecules serve as proton donors for the oxygen‐dependent electron transport chain and the efficient generation of ATP (Eto et al., 1999; Sharma et al., 2005; Stucki, 1976).

Under adverse conditions in which available oxygen is insufficient to meet the energy demands of cells, cytoplasmic pyruvate (the end product of glycolysis) is converted to lactate through anaerobic metabolism which generates ATP in a much less efficient manner. Lactate dehydrogenase (LDH), an NAD^+^/NADH‐dependent enzyme formed by a tetramer of LDHA and LDHB gene products, regulates lactic acid production (Arora et al., 2015; Osis et al., 2021). LDHA isoforms catalyze the conversion of pyruvate to lactate, increasing intracellular lactic acid (Jiang et al., 2021; Osis et al., 2021). In contrast, LDHB drives the opposite reaction converting intracellular lactate to pyruvate (Jiang et al., 2021; Sun et al., 2015). Accordingly, the ratio of LDHA to LDHB subunits determines the overall direction of the pyruvate/lactate conversion. In addition to regulation of lactate production by LDHA and LDHB, lactate itself can be shuttled into and out of cells by the monocarboxylate transporters MCT1 and MCT4, respectively, to impact the acidity of the extracellular environment (Balmaceda‐Aguilera et al., 2012; Whitaker‐Menezes et al., 2011).

In some circumstances, best studied in the context of cancer as the “Warburg effect” or “aerobic glycolysis,” pyruvate is converted to lactate even in the presence of sufficient oxygen. Recently, this phenomenon has been characterized as “metabolic reprogramming” in lung fibroblasts stimulated with the pro‐fibrotic cytokine transforming growth factor beta 1 (TGF‐β1) (Bernard et al., 2015). This metabolic reprogramming has been shown to have a key role in satisfying cellular energy requirements during acute hypoxic stress and has been linked to myofibroblast differentiation and contractility (Aquino‐Gálvez et al., 2019; Bernard et al., 2015; Faulknor et al., 2015; Leinhos et al., 2019).

Published studies have shown increased lactate levels in the lungs from patients with IPF and in the fibrotic lungs of mice following bleomycin injury (Bernard et al., 2015; Kottmann et al., 2012). LDHA expression and LDH activity are increased in fibrotic murine lungs following bleomycin, and inhibition of pyruvate generation with a phosphofructokinase 3B inhibitor or broad‐spectrum LDH inhibition diminished lung fibrosis in that model (Judge et al., 2018; Kottmann et al., 2015; Xie et al., 2015). Consistently, fibroblasts treated with TGF‐β demonstrate increased LDH expression and increased extracellular lactate production (Bernard et al., 2015; Xie et al., 2015). Currently, the metabolic regulation of IPF fibroblasts is not clear. In one study, IPF fibroblasts had an increased extracellular acidification to oxygen consumption ratio (ECAR: OCR), supporting metabolic reprogramming in these cells (Chen, Zhang, et al., 2021; Xie et al., 2015). However, another study showed that compared to age‐matched normal fibroblasts, IPF fibroblasts had decreased ECAR and decreased OCR compared to age‐matched controls (Álvarez et al., 2017). Thus, while fibrotic lungs demonstrate increased lactate levels and pro‐fibrotic mediators stimulate increased lactate in normal fibroblasts, the overall metabolic balance of IPF fibroblasts remains elusive.

Hypoxia, a state of insufficient oxygen delivery to tissues, is a known stimulus of anaerobic respiration and has been implicated in the progression of lung fibrosis (Aquino‐Gálvez et al., 2019; Epstein Shochet et al., 2021; Leinhos et al., 2019; Senavirathna et al., 2018). While hypoxemia (decreased partial pressure of oxygen in the blood) is a common feature in patients with fibrotic lung disease, how fibrotic changes in the alveolar interstitium impact oxygen availability to interstitial fibroblasts, including those within subepithelial fibroblastic foci, are not clear. Supporting the premise that IPF fibroblasts are exposed to chronic hypoxia are studies demonstrating increased HIF‐1α in lungs of bleomycin‐treated rats and in fibrotic tissue of IPF patients (Higgins et al., 2007; Senavirathna et al., 2018). Moreover, several studies support a role of HIF1, and thus chronic hypoxia, in myofibroblast differentiation and tissue fibrosis involving lung, heart, and skin (Modarressi et al., 2010; Wang et al., 2016).

The goal of our study was to examine how hypoxia regulates lactate generation in normal and IPF fibroblasts and to determine whether extracellular lactate itself can regulate fibroblast phenotypes. The studies reported herein demonstrate that hypoxic conditions have differential effects on normal and IPF fibroblasts such that IPF fibroblasts generate increased levels of extracellular lactate through a combination of FoxM1‐mediated increases in LDHA coupled with suppression of LDHB. Moreover, normal fibroblasts exposed to exogenous lactate under conditions of hypoxia differentiate to the myofibroblast phenotype while IPF fibroblasts fail to significantly respond to lactate. Finally, we identify a novel G‐protein‐coupled receptor (GPR‐81) that is induced in control and IPF fibroblasts under hypoxic conditions in a TGF‐β‐independent manner and regulates lactate‐induced myofibroblast differentiation in control fibroblasts. Taken together, our studies support a novel paradigm in which hypoxic conditions induce IPF fibroblasts to produce extracellular lactate which may then function to directly propagate myofibroblast differentiation in “normal” fibroblasts within the same environment.

## 2 MATERIALS AND METHODS

### 2.1 Human subjects and the isolation of primary lung fibroblasts

Lung tissues were removed at the time of transplantation or death from non‐IPF and IPF patients. The tissue samples were stripped of all identifiers and designated as waste (exemption 4). Written informed consent was obtained on all patients or their surrogates prior to the procedure being performed. Use of human lung tissues was approved by the Institutional Review Board (IRB) at the Ohio State University (protocols 2017H0309 and 2021H0180) and all identifiers other than the diagnosis (IPF or “normal”) were removed from the specimens provided. Normal fibroblasts were isolated from the normal lungs of decedents with no evidence of pulmonary disease who were HIV, hepatitis C, and SARS‐CoV‐2 negative and had no evidence of significant trauma. For the preparation of IPF and control lung fibroblasts, the visceral pleura was dissected and removed. The parenchyma was washed with PBS to eliminate RBCs. Minced tissue was transferred to GentleMACs C‐Tubes containing the enzymatic cocktail (Elastase 10 U/mL, Collagenase IV 450 U/mL, Dispase 2 U/mL, and DNAse I 100 μg/mL) and incubated at 37°C. After 1 h, cold serum was added to reduce the enzymatic activity. The filtered lung cell suspension was resuspended in RBC lysis solution, incubated for 7 min, and plated in a 75 cm flask for 24 h. Nonadherent cells were removed, and fresh media was added every 2 days. Experiments were conducted with isolated control and IPF fibroblasts between passages 4 and 7.

### 2.2 Chemical inhibitors, siRNAs, western blot analysis, immunohistochemistry (IHC), and antibodies

2 × 10^5^ control and IPF fibroblasts cultured in the normoxic or hypoxic chambers for 4 days were treated with the GPR‐81 antagonist 3 hydroxy butyric acid (3‐HBA; 10 mM) or TGF‐β (2 ng/mL) for an additional 24 h. For silencing, control and IPF fibroblasts were transfected with 50 nM FoxM1, LDHA, or GPR‐81 siRNA (catalog No. SR320176, SR302665, or SR3223850 from OriGene, respectively) using Lipofectamine 3000 (ThermoFisher, OH, #L3000001). Scrambled siRNA was used as a negative control (catalog No. SR30004). Control and IPF fibroblasts (3 × 10^5^ cells/30 mm cell culture dish) were lysed with 1 × cell lysis buffer (Cell Signaling technology, Beverly, MA) containing protease inhibitor (Roche Applied Science, Indianapolis, IN) and phosphatase inhibitor (Research Products International Corp., Mount Prospect, IL), and western blotting was performed. Cell lysates were collected, sonicated on ice for 15 s, and then denatured using 3× Laemmli buffer at 95°C for 3 min. Protein quantification was conducted by *DC™* Protein Assay Kit I (Bio‐Rad Laboratories, CA, #5000111). Protein samples (5 to 15 μg) were loaded and separated through the gradient polyacrylamide gels (Novex WedgeWell Tris‐Glycine Gel) having 4%–20% polyacrylamide concentration (Invitrogen, CA, #XP04205). Precision Plus Protein Kaleidoscope Prestained Protein Standards (Bio‐Rad, #1610375) were used as a protein marker. Proteins on the gel were then electrically transferred to a PVDF membrane (Bio‐Rad, #1620177) using a Protean III tank transfer system (Bio‐Rad). After blocking with 5% bovine serum albumin in 1× TBST (0.1 M Tris, 0.9% NaCl, and 0.1% Tween 20) for 1 h at room temperature, the membrane was incubated with antibodies (1:1000 dilution in 1x TBST containing 1% bovine serum albumin) as follows: FoxM1 (Cell signaling, MA #20459, AB_2798842, Im et al., 2018), LDHA (Cell signaling, #2012, AB_2137173, Feng et al., 2022), LDHB (ThermoFisher, 14824‐1‐AP, AB_2134953, Izquierdo‐Álvarez et al., 2012), GPR‐81 (ThermoFisher, PA5‐114741 AB_2899377), α‐smooth muscle actin (α‐SMA, Cell signaling #14968 AB_2798667, Yao et al., 2021), GAPDH (Cell signaling, #2118, AB_561053, Wang et al., 2021), HIF1α (proteinintech, IL, #20960‐1‐AP, Qian et al., 2019), and Goat anti‐Rabbit IgG secondary antibody (ThermoFisher, #32460, AB_1185567). The protein bands were detected by ECL solution (ThermoFisher Scientific, #32106), and images were obtained with an image analyzer using Image Lab™ Touch Software (Bio‐Rad). Protein band density was quantified and analyzed by ImageJ (win 64).

Immunohistochemical analysis for the lung tissues derived from non‐IPF and IPF patients was also performed. Briefly, human lung tissues embedded in paraffin were cut at 7 μm thickness and mounted onto polylysine‐coated slides. The sections were deparaffinized in xylene, rehydrated through a graded series of methanol, and placed in a water bath set at 98°C for 20 min in citrate buffer (pH 6.0) for antigen retrieval. All slides were then transferred to a humid staining rack in distilled water. All tissue on slides were circled with an immunopen, and then two drops of TBS were added to each slide and left to sit for 5 min. Endogenous peroxidases were quenched with 3% hydrogen peroxide in PBS for 5 min and sections were incubated with diluted normal swine serum to block nonspecific binding of secondary antibodies for 15 min at room temperature. The slides were incubated overnight at 4°C with 1:200 dilution of LDHB (Prointech, IL, #14824‐1‐AP, AB_2134953) or 1:100 dilution of GPR‐81 (ThermoFisher, #PA5‐114741, AB_2899377). After incubation, the slides were washed with PBS and then incubated with goat anti‐rabbit biotin‐conjugated secondary antibody (MilliporeSigma, #AP132B, Burlington, MA, USA) for 60 min (1:1500 dilution). After washing with TBS, the slides were incubated in HRP‐Streptavidin solution (Vector Laboratories) for 45 min and subjected to the application of the chromogen substrate 3, 3′ Diaminobenzene for 3 min and counterstained with hematoxylin. Slides were then put into a water bath with running tap water and then were rinsed in PBS before being dehydrated through a graded series of ethanol. Slides were then put into xylene before being treated with permanent mounting medium and coverslips being applied. Slides were then left to dry overnight. Images were obtained using a EVOS M7000 microscope (Invitrogen) and processed with Celleste Image analysis version 5.0 software. Control specimens were processed under the same conditions without the primary antibody to produce negative control (NC) sections.

### 2.3 Hypoxic chamber, cell viability, and lactate assay

Control and IPF fibroblasts were cultured at 37°C in a modular hypoxic chamber (CellXpert, C170i, Eppendorf, Enfield, CT) flushed with a gas mixture containing 1% O_2_, 5% CO_2_, and 94% N_2_. Control and IPF fibroblasts (2 × 10^4^ cells/well of a 96‐well plate) were cultured in serum containing DMEM medium under the normoxic and hypoxic conditions for 5 days. To minimize the potential for reoxygenation, the cells were cultured in the hypoxic conditions without interruption. To precisely measure extracellular lactate levels, control and IPF fibroblasts were incubated in the hypoxic chamber for 5 days without changing the medium. For the measurement of cell viability, control and IPF fibroblasts cultured under normoxic and hypoxic conditions as indicated were incubated with 20 μL of Cell Titer Blue reagent (Promega, WI) for 3 h, and cell viability was measured at 560 nm (Ex)/590 nm (Em) of fluorescence using a 96‐well plate reader (BioTek, VT). Extracellular lactate levels were measured using a Lactate Assay kit (Millipore Sigma, MO). Briefly, extracellular medium derived from control and IPF fibroblasts cultured under the conditions as described above was collected. Lactate standard was prepared and the final concentration of lactate (ng/μl) was measured according to the manufacturer's protocol.

### 2.4 Statistics

Data are expressed as the means ± SD. Two‐dimensional column graphs were prepared using Microsoft Excel. Protein expression levels are also presented as a box‐whisker plot showing the lowest expression, lower quartile, median, upper quartile, and the highest expression using SPSS v.19. N equals the number of unique and independent experimental samples. The significance of the sample data was determined using student *t* test (two‐tailed), and two sample equal variance was used. Significance level was set at *p* < 0.05.

## 3 RESULTS

### 3.1 Extracellular lactate production is increased in IPF fibroblasts under hypoxic conditions

**FIGURE 1:**
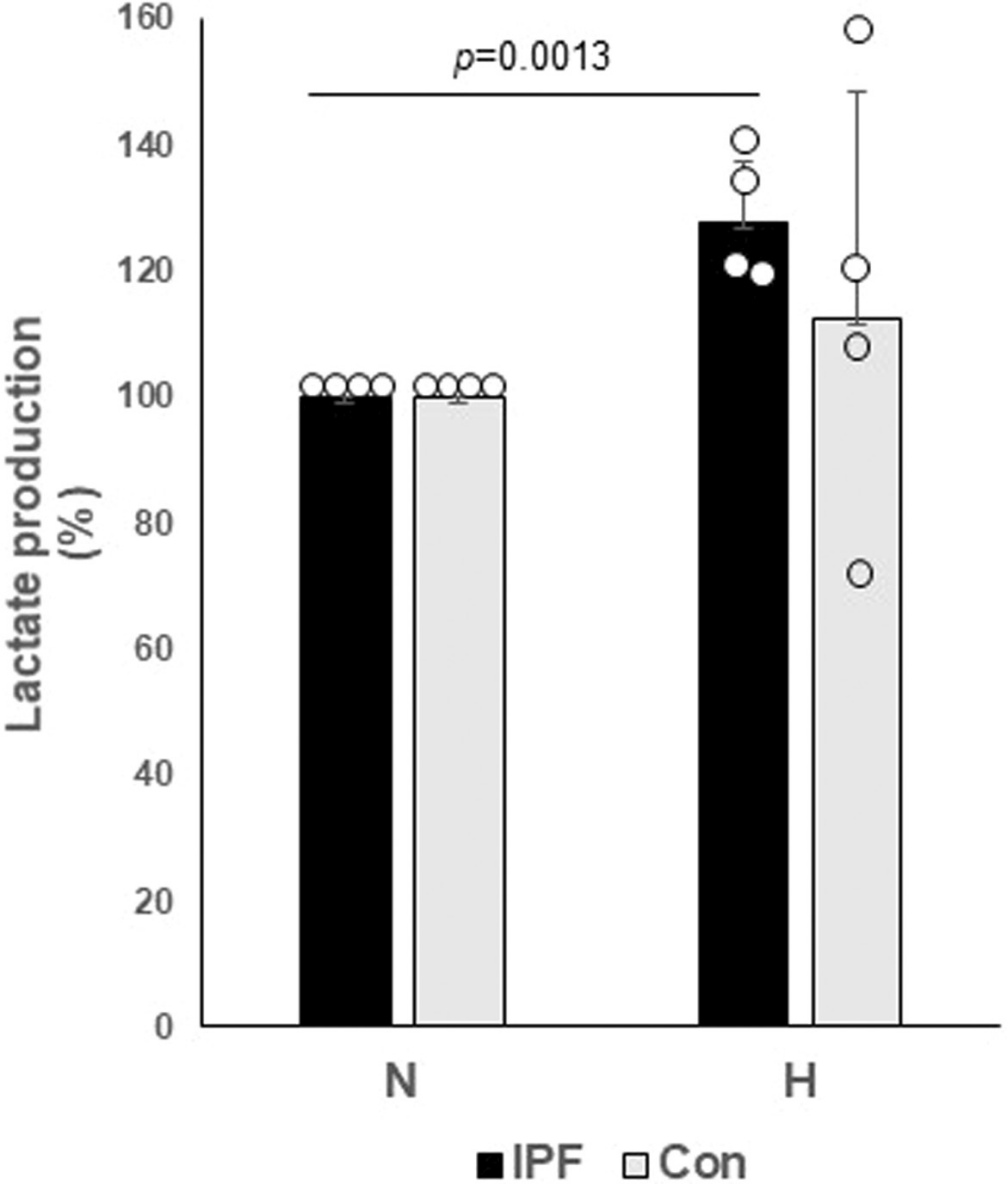
Control and IPF fibroblasts (*n* = 4, each) were cultured in normoxic (N) and hypoxic (H, 1% oxygen) conditions for 5 days in serum containing DMEM medium, and extracellular lactate concentrations (ng/μL) were measured. The change in extracellular lactate levels under hypoxic conditions expressed as percentage of basal lactate production in the same cohort of cells under normoxic conditions (expressed as 100%) is shown. Each dot represents a separate control or IPF fibroblast. **p = 0.0013 compared to lactate levels from IPF fibroblasts under normoxic conditions.

Prior studies have linked hypoxia to the pathogenesis of fibrotic diseases including lung fibrosis (Senavirathna et al., 2018; Short et al., 2004). Hypoxia stimulates a shift from normal mitochondrial respiration toward anaerobic respiration, and the resulting increase in extracellular lactate has also been implicated in fibrosis (Higgins et al., 2007; Judge et al., 2018; Senavirathna et al., 2018; Watanabe et al., 2008). While IPF fibroblasts have altered metabolism under conditions of hypoxia, there is a gap in our understanding of how responses to hypoxia are regulated in normal and IPF fibroblasts. To address this gap, control (nonfibrotic) and IPF fibroblasts were cultured under persistent normoxic and hypoxic (1% oxygen for 5 days) conditions and extracellular lactate levels were measured. HIF‐1α is a molecular sensor for hypoxia and increased in response to oxygen deficiency (Epstein Shochet et al., 2021; Short et al., 2004). Thus, we first measured HIF‐1α levels in control fibroblasts cultured under hypoxic conditions. Confirming the role of HIF‐1α as responsive to our conditions in normal fibroblasts, HIF‐1α levels were significantly increased by hypoxia (Supporting Information Figure S1). We next measured extracellular lactate and observed similar levels in control and IPF fibroblasts cultured under normoxic conditions (Supporting Information Figure S2). When exposed to hypoxia, extracellular lactate levels increased in both cell populations. However, the relative increase in lactate levels were more pronounced in the IPF fibroblasts (Figure 1). These findings suggest that when oxygen is not sufficiently available, a lactate‐rich environment is generated by IPF fibroblasts.

### 3.2 Skewed regulation of LDHA and LDHB expression under hypoxic conditions accentuates lactate production in IPF fibroblasts

**FIGURE 2:**
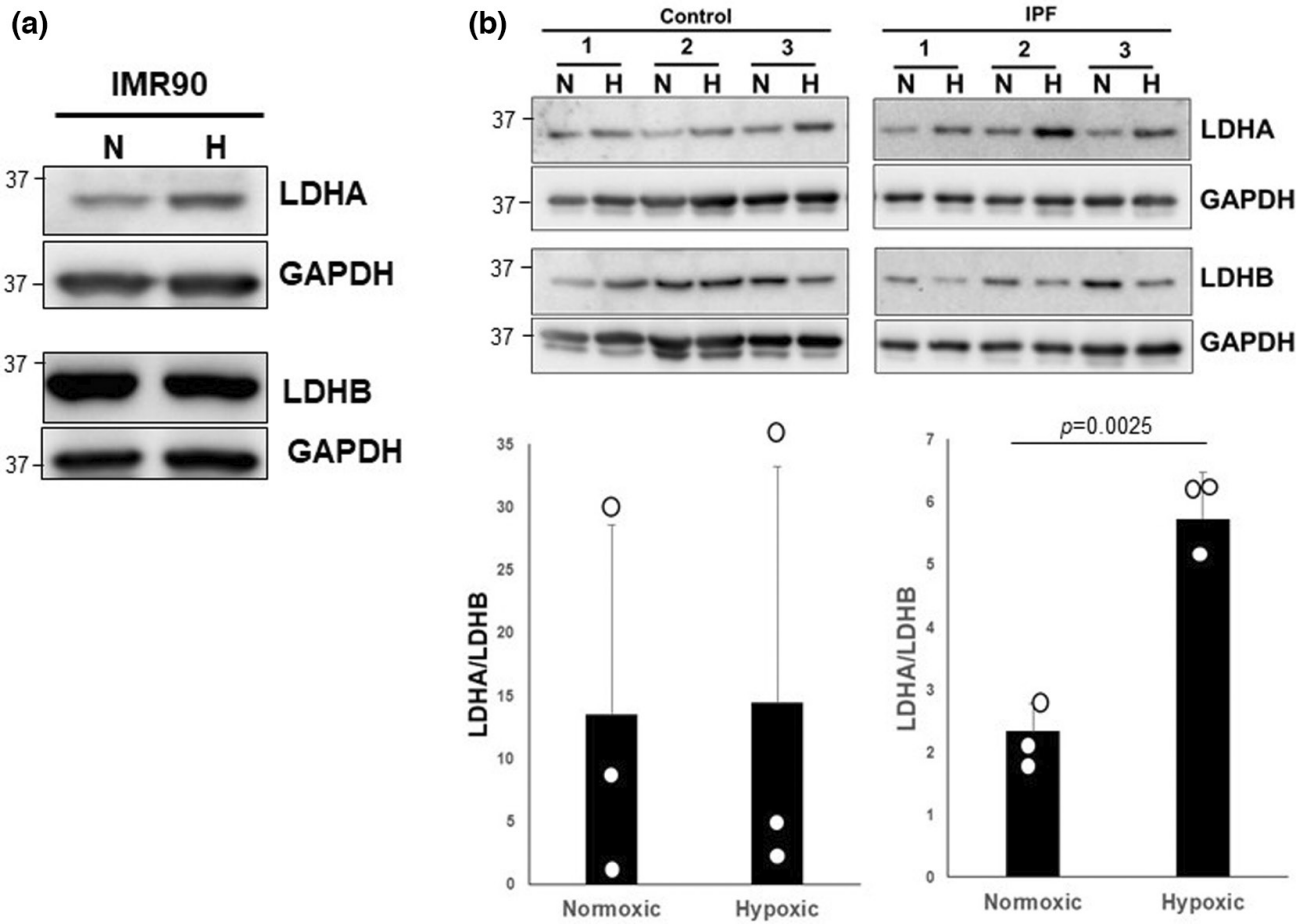
(a) IMR‐90 cells were cultured in normoxic (N) or hypoxic (H) conditions for 5 days, and LDHA and LDHB levels were measured. (b) *Upper*, Control and IPF fibroblasts (*n* = 3 distinct cell lines each) were cultured under the same conditions and LDHA and LDHB protein levels were determined by western blot. GAPDH was used as a loading control. *Lower*, Quantification of the LDHA/LDHB ratio in Control and IPF fibroblasts was determined by densitometry and expressed by a box‐whisker plot; LDHA and LDHB levels from each control and IPF fibroblast line are shown in the supplemental data section. **p = 0.0025 compared to normoxic condition.

Five isoforms of NADH/NAD^+^‐dependent lactate dehydrogenase (LDH) regulate the interconversion between pyruvate and lactate (Kottmann et al., 2015). Among them, LDHA converts pyruvate to lactate, while LDHB has a higher affinity for lactate which it oxidizes to regenerate pyruvate (Kottmann et al., 2015). Thus, a high LDHA/LDHB ratio favors increased lactate production from pyruvate. To determine whether hypoxia‐mediated lactate generation in IPF fibroblasts was regulated by the abnormal equilibrium of LDHA and LDHB, we first examined these proteins in IMR‐90 fibroblasts under normoxic and hypoxic conditions. In this line of primary normal fetal lung fibroblasts, LDHA levels were increased by hypoxic conditions while LDHB was not significantly changed (Figure 2a). Because IMR‐90 fibroblasts are derived from fetal lungs and aging contributes to the development of lung fibrosis, we next examined LDHA and LDHB expression in age‐matched control and IPF fibroblasts under normoxic and hypoxic conditions. Compared to normoxia, LDHA/LDHB ratios were heterogeneously, but not significantly impacted by hypoxia in control fibroblasts (Figure 2b, upper and lower left, Supporting Information Figures S3 and S4). Similarly, LDHB levels were heterogeneously impacted by hypoxia in the control cell lines, but on average were not significantly altered by hypoxia (Figure 2b, upper left). In contrast to the control fibroblasts, IPF fibroblasts exposed to hypoxia showed consistently robust increases in LDHA levels that were coupled with a clear decline LDHB expression (Figure 2b, upper right, Supporting Information Figures S3 and S4). This led to a significant augmentation in the LDHA/LDHB expression ratio (Figure 2b, lower right). This finding supports a skewed equilibrium of LDHA/LDHB in IPF fibroblasts that is consistent with the accentuated extracellular lactic acid observed in the IPF fibroblasts under hypoxic conditions.

### 3.3 FoxM1 regulates LDHA in response to hypoxia

**FIGURE 3:**
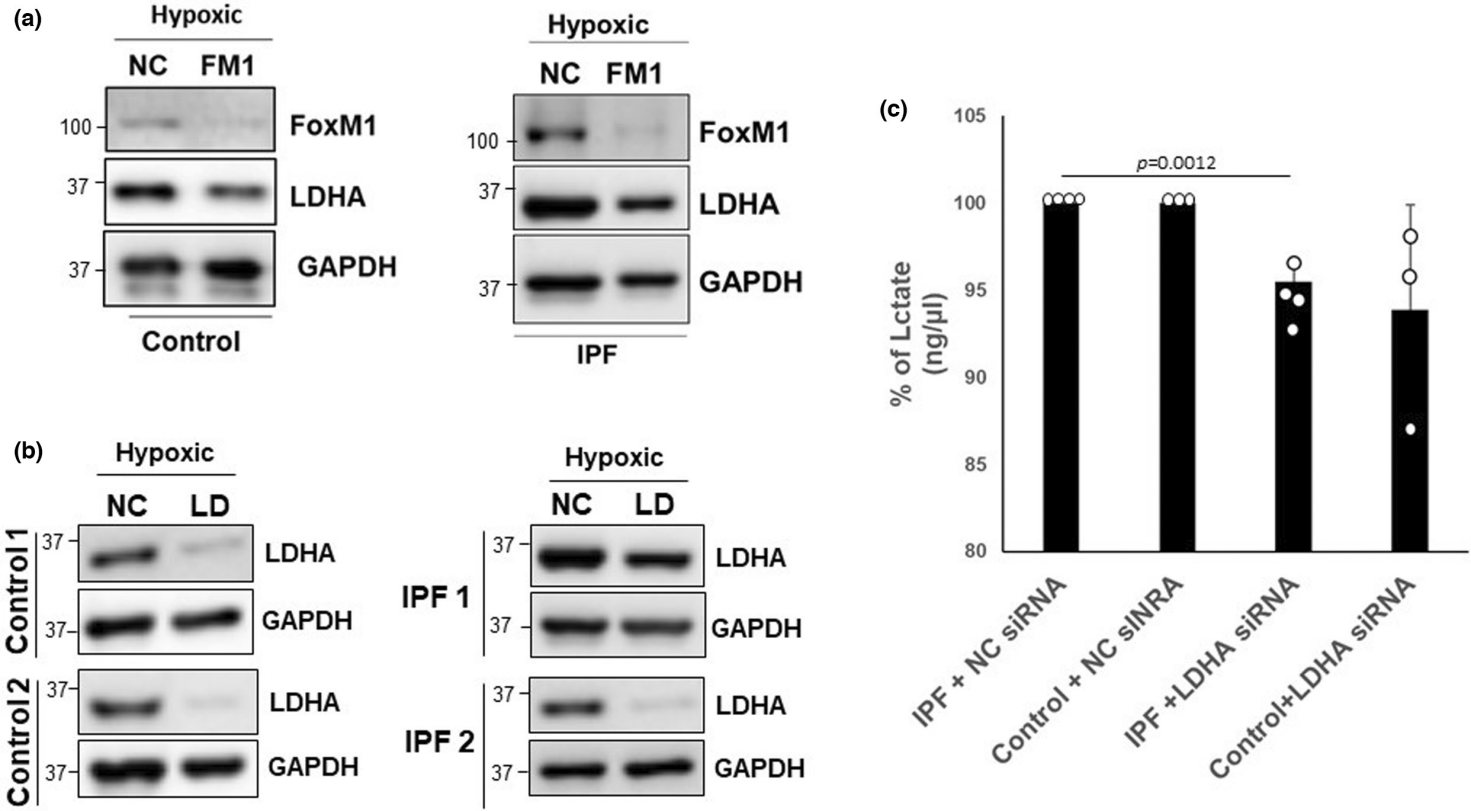
(a) Representative images of FoxM1 and LDHA levels of IPF or control fibroblasts cultured under hypoxic conditions for 3 days followed by the treatment with 50 nM of either negative control (NC) or FoxM1 (FM1) siRNA for additional 2 days. GAPDH was used as a loading control. (b) Representative images of LDHA levels of control (*n* = 3) or IPF fibroblasts (*n* = 3) cultured under the hypoxic conditions for 3 days followed by the treatment with 50 nM of negative control (NC) or LDHA (LD) siRNA duplex for additional 2 days. (c) Extracellular lactate concentration (ng/μl) in control (*n* = 3) and IPF cells (*n* = 4) were measured under the same conditions. The change (%) of lactate in the presence or absence of LDHA siRNA compared to baseline levels under hypoxic conditions (100%) is shown. **p = 0.0012 compared to lactate levels from NC siRNA transfected IPF fibroblasts.

FoxM1 is reported to be a transcriptional regulator of LDHA (Wang et al., 2016), and FoxM1 is aberrantly regulated in IPF fibroblasts (Im et al., 2018). To directly assess the role of FoxM1 in the regulation of LDHA, we silenced FoxM1 using dicer‐substrate siRNAs in control and IPF fibroblasts and measured LDHA expression. FoxM1 levels were clearly reduced when control or IPF cells were transfected with the targeting siRNA. Silencing of FoxM1 in both cell types was also associated with a significant decrease in LDHA expression under hypoxic conditions (Figure 3a). However, FoxM1 silencing did not affect LDHB expression in control and IPF cells (Supporting Information Figure S5), indicating that LDHA is the direct target of FoxM1 and that there is differential regulation of LDHA and LDHB transcription. To further test the role of LDHA on lactate expression, we then silenced LDHA in control and IPF fibroblasts (Figure 3b) and measured extracellular lactate levels under hypoxic conditions. Consistently, in response to silencing LDHA under hypoxic conditions, extracellular lactate in the IPF fibroblasts was suppressed to levels comparable to control fibroblasts (Figure 3c). Collectively, these data link pro‐fibrotic FoxM1 to the regulation of LDHA and extracellular lactate production induced by hypoxia in IPF fibroblasts.

### 3.4 TGF‐β does not regulate LDHA or LDHB in fibroblasts under hypoxic conditions

**FIGURE 4:**
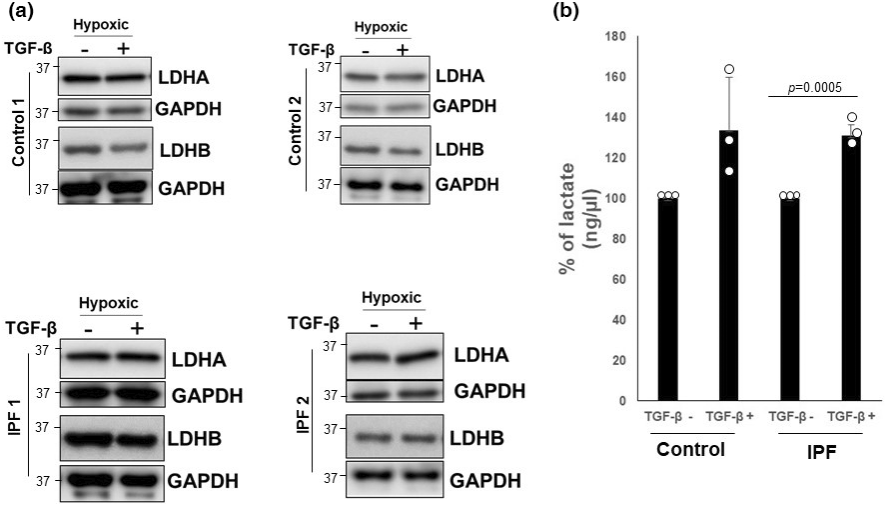
(a) Control (*n* = 3) and IPF fibroblasts (*n* = 3) were cultured under hypoxic conditions for 4 days and for additional 16 h in the presence or absence of TGF‐β (2 ng/mL). representative α‐SMA, LDHA, and LDHB protein expression in controls (upper) and IPF (lower) are shown. (b) Control and IPF fibroblasts (*n* = 3) were cultured in the presence or absence of TGF‐β (2 ng/mL) in the hypoxic chamber. Extracellular lactate concentrations (ng/μL) were measured and expressed as the percentage change compared to baseline in the absence of TGF‐β (100%). ***p = 0.0005.

TGF‐β is a pro‐fibrotic cytokine implicated in the differentiation, metabolic reprogramming, and increased extracellular lactic acid in normal lung fibroblasts (Bernard et al., 2015; Kottmann et al., 2012). To test the role of TGF‐β on the regulation of lactate expression under oxygen deprived conditions, we examined the effect of exogenous activated TGF‐β on LDHA and LDHB protein expression in response to hypoxia. Under conditions of hypoxia, there was no significant change in LDHA or LDHB levels in the control fibroblasts (Figure 4a, upper) or the IPF fibroblasts (Figure 4a. lower). We next measured extracellular lactate levels in control and IPF fibroblasts under hypoxic conditions in the presence or absence of TGF‐β. Although the absolute levels of lactate generation were elevated in both the control and IPF fibroblasts under hypoxic conditions (Supporting Information Figure S6), there was a more consistent and significant increase in lactate in the IPF fibroblasts in response to TGF‐β treatment combined with hypoxia (Figure 4b). Collectively, these results show that hypoxia promotes extracellular lactate generation through the modulation of LDHA (control and IPF fibroblasts) and LDHB (IPF fibroblasts). Moreover, under hypoxic conditions, TGF‐β can further stimulate increased extracellular lactate through LDHA/LDHB‐independent mechanisms, suggesting that TGF‐β may enhance extracellular lactate levels through additional mechanisms.

### 3.5 Hypoxia suppresses α‐SMA expression in normal, but not IPF fibroblasts

**FIGURE 5:**
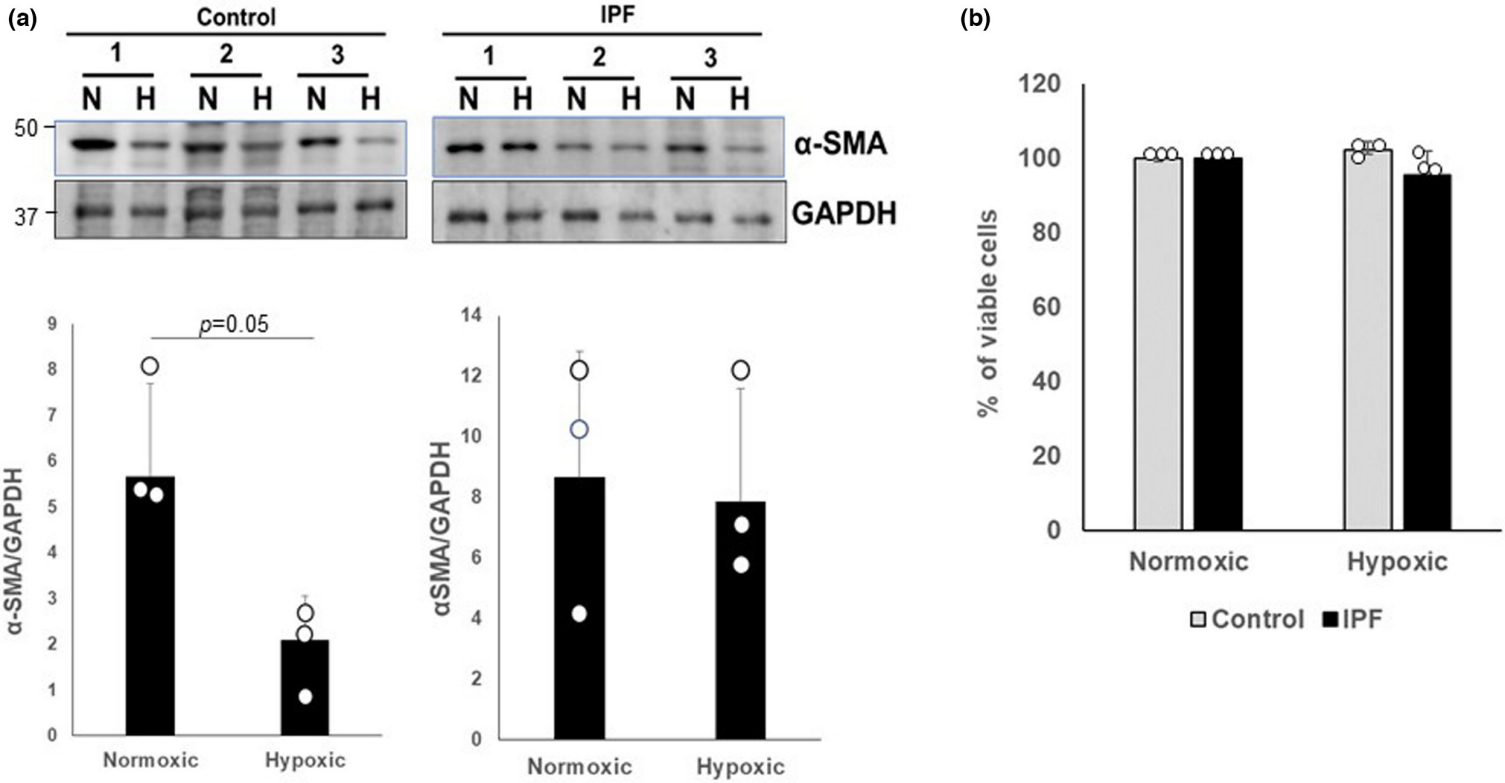
(a) Control (left, *n* = 3) and IPF fibroblasts (right, *n* = 3) were cultured under normoxic (N) and hypoxic conditions for 5 days. α‐SMA levels were then measured. GAPDH was used as a loading control and band density measured and presented (lower). *p = 0.05. (b) The percentage of viable control and IPF fibroblasts cultured under normoxic and hypoxic conditions for 5 days, with the normoxic controls set at 100% is shown.

Prior reports indicate that hypoxia can induce fibroblast differentiation in normal fibroblasts (Chen, Zhang, et al., 2021; Kottmann et al., 2015). These findings suggest that α‐SMA expression is modulated when control fibroblasts are exposed to hypoxic conditions. We next examined the impact of persistent hypoxia on fibroblast differentiation and found that α‐SMA expression significantly decreased (Figure 5a, upper and lower left). In contrast, α‐SMA expression remained stable in IPF fibroblasts exposed to the same persistent hypoxic conditions (Figure 5a, upper and lower right). To ensure that the observed differences in α‐SMA expression with hypoxia were not due to differential induction of cell death, we additionally assessed fibroblast viability. Under our experimental conditions, we did not observe any significant impact of hypoxia on viability of control or IPF fibroblasts (Figure 5b). These results indicate that hypoxia diminishes differentiation in normal fibroblasts while fibrotic fibroblasts maintain a stable differentiated phenotype.

### 3.6 Exogenous lactate increases α‐SMA under hypoxic, but not normoxic conditions

**FIGURE 6:**
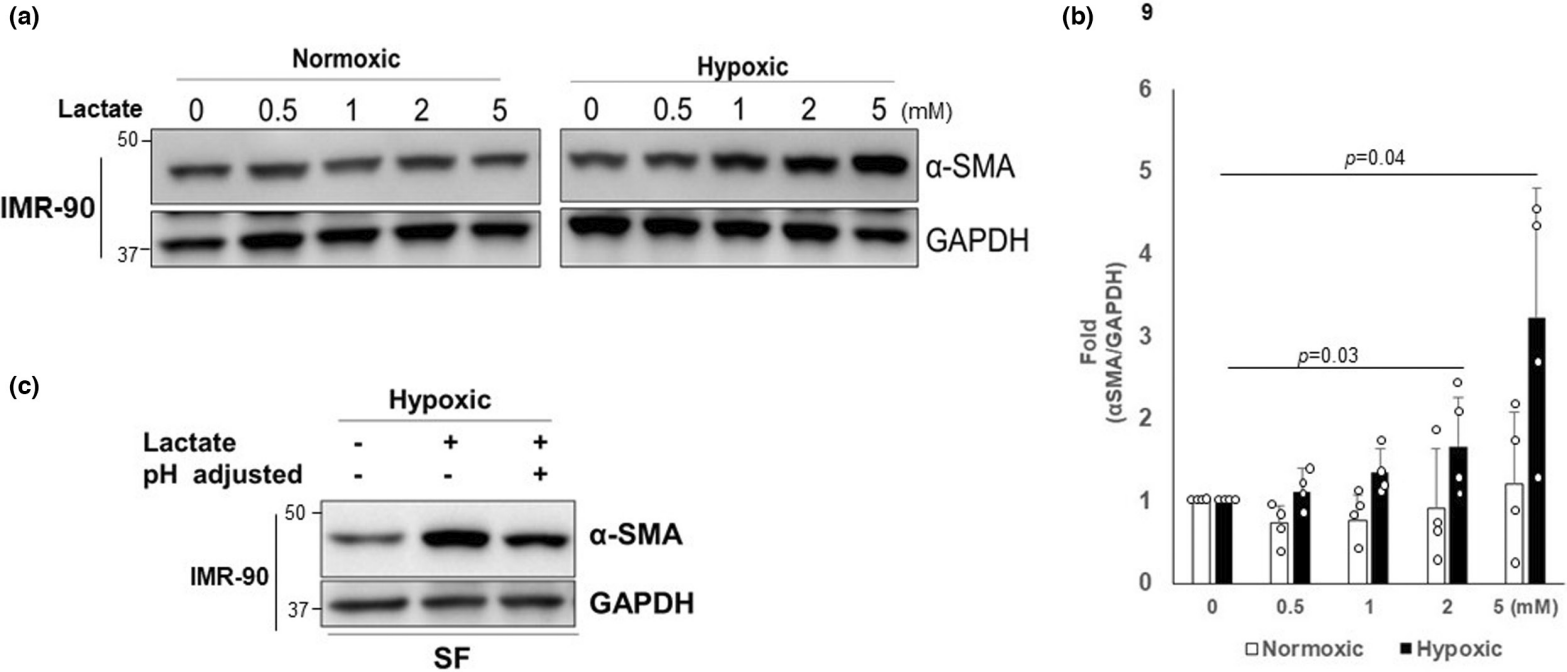
(a) IMR‐90 cells treated with lactate ranging from 0.5 to 5 mM were cultured under normoxic or hypoxic conditions for 5 days. Α‐SMA levels were then measured. Blots were reprobed for GAPDH. (b) The α‐SMA/GAPDH protein levels under normoxic and hypoxic conditions in response to various doses of lactate is shown. α‐SMA/GAPDH protein levels of non‐treated control or IPF fibroblasts set at 1, and fold‐change with lactate treatment was determined. Data shown represent three independent experiments. *p = 0.05 compared to α‐SMA level in the absence of lactate treatment. (c) Serum‐free DMEM media (SF) was either adjusted to a pH of 7.3 or not adjusted, and IMR‐90 cells were cultured with or without lactate for 5 days in the hypoxic chamber. α‐SMA levels were then measured.

Our studies show that IPF fibroblasts generate increased lactate and have a stable differentiated phenotype under hypoxic conditions, while control fibroblasts, under the same conditions, generate lower levels of extracellular lactate and have reduced α‐SMA expression. These findings prompted us to hypothesize that extracellular lactate may have a direct effect on the differentiation state of fibroblasts. To test this, we exposed IMR‐90 fibroblasts to various doses of exogenous lactate under normoxic and hypoxic conditions and assessed α‐SMA expression. α‐SMA levels remained stable in response to various doses of lactate under normoxic conditions (Figure 6a, left panel and 6B open bars). However, in hypoxic conditions, α‐SMA levels increased in a dose‐responsive manner (Figure 6a, right panel and 6B black bars), supporting a paradigm in which extracellular lactate itself can induce differentiation of normal fibroblasts under oxygen‐deprived conditions.

Prior reports have indicated that a low pH can induce myofibroblast differentiation by liberating latent TGF‐β stored in the extracellular matrix or by activating TGF‐β in serum (Kottmann et al., 2012). To test this possibility, fibroblasts cultured in serum‐free media under hypoxic conditions were treated with exogenous lactate in media with pH 7.3 (nonacidic, pH adjusted) or in media in which the pH was not adjusted. Notably, treatment with exogenous lactate under nonacidic conditions (pH adjusted) also increased α‐SMA expression in control fibroblasts, although the extent of a‐SMA expression was reduced compared to cells treated without pH adjustment (Figure 6c, lane 3). This demonstrates a direct pH‐independent effect of lactate on the normal fibroblasts differentiation that can be further amplified in the presence of an acidic pH.

### 3.7 Hypoxia increases GPR‐81 expression in normal and IPF fibroblasts

**FIGURE 7:**
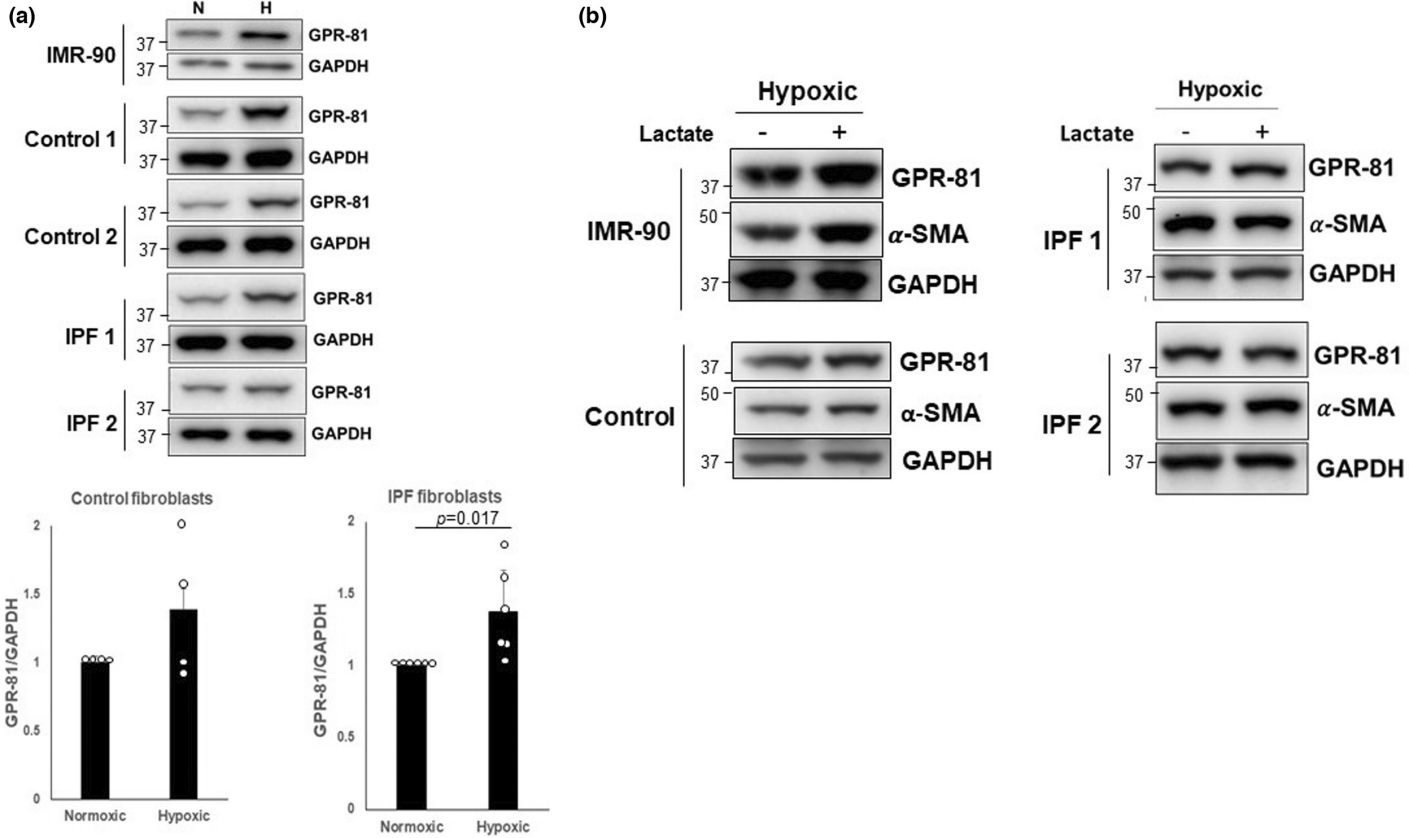
(a) *Upper*, IMR‐90, control and IPF fibroblasts were cultured under normoxic (N) and hypoxic (H) conditions for 5 days, and GPR‐81 was measured by western blot. Representative blots from IMR‐90, control (*n* = 4), and IPF fibroblasts (*n* = 6, each). *Lower*, GPR‐81/GAPDH levels in control and IPF fibroblasts under normoxic and hypoxic conditions. Shown is the fold change of GPR‐81 levels in control and IPF fibroblasts under hypoxic condition compared to that of normoxic condition set as 1. *p = 0.017. (b) *Left*, IMR‐90 and control fibroblasts (*n* = 3) treated with 5 mM lactate were cultured under hypoxic conditions for 5 days. α‐SMA and GPR‐81 levels were then measured. *Right*, IPF fibroblasts (*n* = 3) treated with 5 mM lactate were cultured for 5 days under hypoxic conditions. α‐SMA and GPR‐81 levels were measured. GAPDH was used as a loading control.

A variety of G protein‐coupled receptors (GPRs) have been implicated in the pathogenesis of pulmonary fibrosis (Choi et al., 2021; Haak et al., 2020). GPR‐81 is a cognate receptor for lactate that is expressed in multiple cell types. To test its role in the regulation of fibroblast phenotype by extracellular lactate, we examined GPR‐81 expression in control and IPF fibroblasts under normoxic and hypoxic conditions and found that hypoxia increased GPR‐81 in both control and IPF fibroblasts (Figure 7a, upper and lower). In normal IMR‐90 fibroblasts under hypoxic conditions, lactate itself induced increased expression of GPR‐81 which was associated with increased α‐SMA expression (Figure 7b, left). In control fibroblasts, GPR‐81 and α‐SMA levels were also moderately or slightly increased in response to hypoxic conditions. In contrast, neither GPR‐81 nor α‐SMA was significantly increased in IPF fibroblasts treated with lactate under hypoxic conditions (Figure 7b, right). These results suggest a potential novel mechanism in which lactate induces myofibroblast differentiation in control fibroblasts under oxygen‐deprived conditions. In contrast, the lack of GPR‐81 induction and myofibroblast differentiation following lactate exposure in IPF fibroblasts when oxygen is deficient suggests an ability of IPF fibroblasts to evade these microenvironmental cues.

**FIGURE 8:**
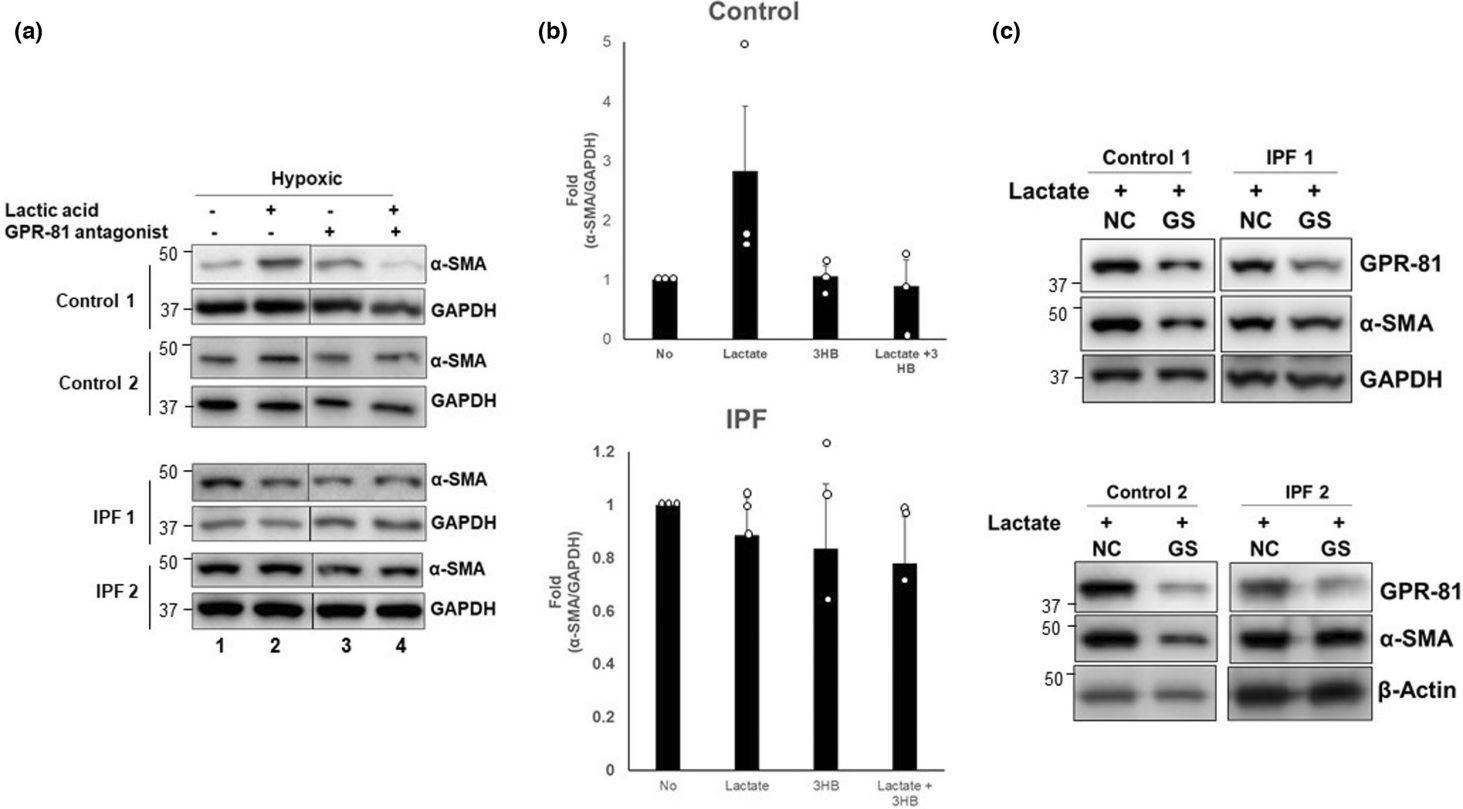
(a) Representative α‐SMA protein levels in control and IPF fibroblasts (*n* = 3) treated with 5 mM of lactate and/or 1 mM of 3 hydroxybutyric acid incubated in the hypoxic chamber for 5 days. GPAPDH was used as a loading control. Vertical lines indicate that blot images are from different lanes of the same membranes. (b) The α‐SMA/GAPDH levels in control (*upper*) and IPF fibroblasts (*lower*). α‐SMA/GAPDH levels of non‐treated (No) control or IPF fibroblasts were set as 1, and fold‐change compared to that baseline are shown. 3HB: 3 hydroxybutyric acid. (c) Control and IPF fibroblasts (*n* = 3) transfected with 50 nM of negative control (NC) or GPR‐81 siRNA (GS) were cultured under normoxic condition for 48 h. Cells were then treated with lactate (50 mM) and cultured for 24 h under hypoxic condition. GPR‐81 and α‐SMA levels were then measured.

To further evaluate the role of GPR‐81 in lactate‐mediated myofibroblast differentiation under hypoxic conditions, we next treated control and IPF fibroblasts with lactate in the presence or absence of the GPR‐81 antagonist, 3‐hydroxybutyric acid (Chen, Zhou, et al., 2021; Shen et al., 2015). When control fibroblasts were cultured in the presence of lactate, α‐SMA levels were increased (Figure 8a, lane 2, Figure 8b, upper). However, this induction of α‐SMA was blocked in the presence of the GPR‐81 antagonist (lane 4). Consistent with our prior findings, lactate had no major effect on α‐SMA expression in IPF fibroblasts under hypoxic conditions, and the GPR‐81 antagonist similarly had no significant impact (Figure 8a, lower, lanes 2 and 4, Figure 8b, lower). These results support a GPR‐81‐mediated pro‐fibrotic effect of lactate on normal, but not IPF lung fibroblasts under oxygen‐deprived conditions. To confirm this, we transfected control and IPF lung fibroblasts with siRNA targeting GPR‐81, and α‐SMA levels were examined under the hypoxic conditions. Like the findings with the GPR‐81 antagonist, GPR‐81 silencing clearly reduced α‐SMA levels in control fibroblasts while the levels of α‐SMA remained unaltered in IPF fibroblasts under the same conditions (Figure 8c, upper and lower). Taken together, these findings suggest that the cellular effect of lactate that promotes myofibroblast differentiation is mainly seen on control fibroblasts, and that extracellular lactate produced by IPF fibroblasts may function to directly increase differentiation in neighboring normal lung fibroblasts.

**FIGURE 9:**
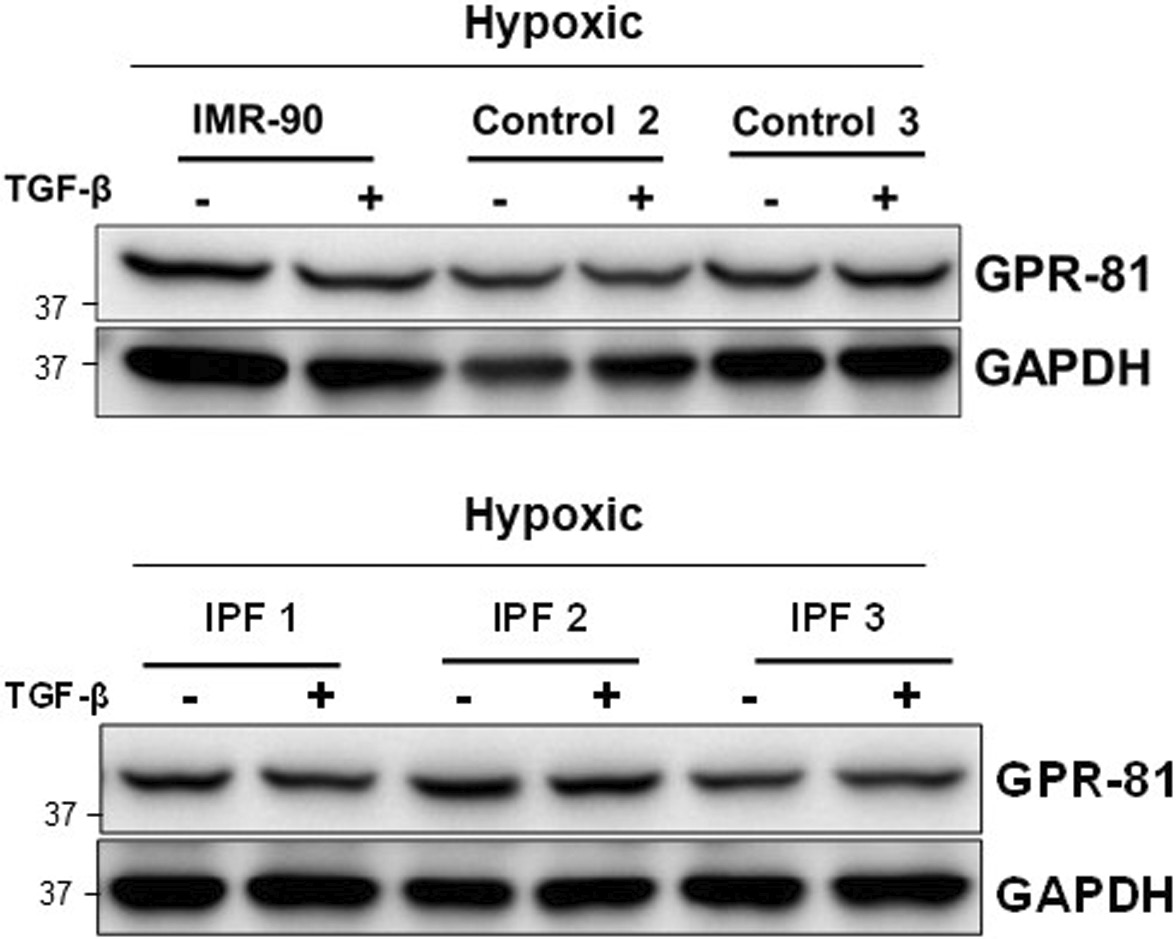
(a) IMR‐90, control (*n* = 2), and IPF fibroblasts (*n* = 3) were cultured for 4 days in the hypoxic chamber. Cells were then incubated in the presence or absence of TGF‐β (2 ng/mL) for additional 24 h. GPR‐81 levels were then measured. GAPDH was used as a loading control.

Given the prominent role of TGF‐β in fibrosis, we further examined whether TGF‐β regulated GPR‐81 levels in control and IPF fibroblasts under hypoxic conditions. GPR‐81 levels were unaltered by TGF‐β (Figure 9), further indicating that hypoxia, and not TGF‐β, is responsible for the upregulation of a lactate receptor and lactate/GPR‐81‐dependent myofibroblast differentiation.

### 3.8 Enhanced GPR‐81 and reduced LDHB expression is found in IPF fibroblast patient tissues

**FIGURE 10:**
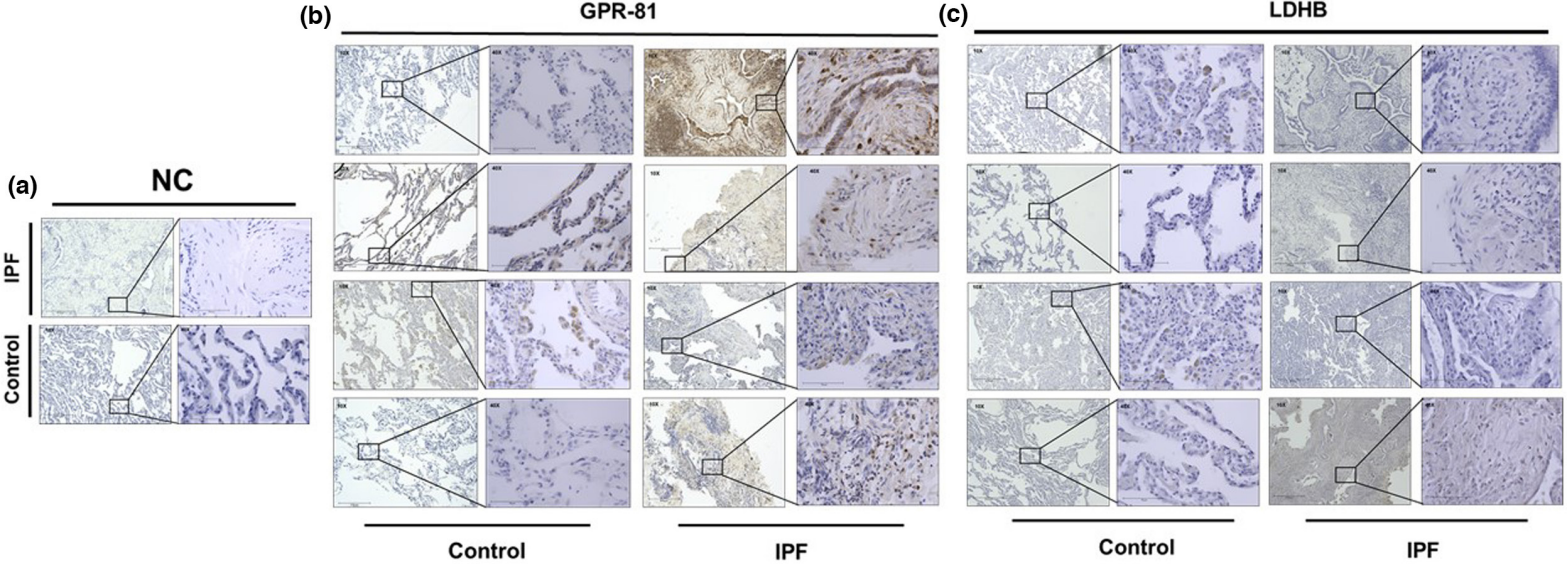
Immunohistochemistry of non‐IPF (control) and IPF lung tissues (*n* = 4, respectively). (a) Negative control IHC images of non‐IPF (control) and IPF lung tissues (NC). (b) GPR‐81 expression in control and IPF lung tissues. (c) LDHB in control and IPF lung tissues. Left and right slides show 10× and 40× images, respectively. Bar indicates the size of tissues (10×: 275 μm, 40×: 75 μm). Boxes show the locations of 40× magnified images in 10× magnifications.

A prior study showed the enhanced LDHA expression in IPF patient lung tissues (Kottmann et al., 2012). Our results showed that IPF fibroblasts expressed enhanced GPR‐81 and reduced LDHB under hypoxic conditions. Thus, we next examined GPR‐81 and LDHB expression in the lung tissues from IPF and non‐IPF patients. Enhanced GPR‐81 expression was found in IPF patient tissues compared to that from non‐IPF patients (Figure 10b, IPF). The increased expression of GPR‐81 was found in the myofibroblasts located in the fibroblastic foci as well as lung epithelium overlying the fibroblastic foci. Unlike this, compared to non‐IPF lung tissues, LDHB expression was low or almost absent in IPF lung tissues including the fibroblastic foci (Figure 10c, IPF). Collectively, prior results and our new findings suggest that GPR‐81, LDHA, and LDHB were also abnormally expressed in IPF lung tissues, further supporting our concept that the alteration of LDHA/LDHB and lactate/GPR‐81‐mediated signaling may contribute to the pathobiology of IPF.

## 4 DISCUSSION

Metabolic alterations have been linked to various human diseases including lung fibrosis. In cancer cells, aerobic glycolysis (the Warburg effect) is known to be activated leading to increased production of lactate despite an ample supply of oxygen (Devic, 2016). In lung fibrosis, remodeling of the distal airway and alveolar interstitium impedes oxygen diffusion from the alveolar space through the epithelium, the interstitium, the endothelium, and into blood resulting in hypoxemia. The increased expression of HIF‐1α supports the premise that fibroblasts within fibroblastic foci and the fibrotic interstitium of IPF tissue are subjected to cellular hypoxia due to impaired diffusion (Higgins et al., 2007). Whether due to metabolic reprogramming and aerobic glycolysis or to impaired oxygen diffusion to fibroblasts in the alveolar interstitium, elevated lactic acid levels have been found in lung tissues from IPF patients (Bell et al., 2022; Kottmann et al., 2012). However, the precise mechanism(s) driving lactate generation in fibrotic lung fibroblasts and the potential role of lactate‐induced cellular signaling on fibroblast phenotype are poorly understood. We found that lactate generation in fibrotic lung fibroblasts is not driven solely by increased LDHA, as previously described (Kottmann et al., 2012), but by a concomitant reduction in LDHB leading to an amplified skewing of the LDHA/LDHB ratio. Moreover, we demonstrate that lactate itself can promote myofibroblast differentiation in normal lung fibroblasts through the GPR‐81 lactate receptor. Our IHC analysis with IPF patient lung tissues also showed that GPR‐81 and LDHB were abnormally expressed in patients with IPF, with robust GPR‐81 in the fibroblastic foci, the interstitium, and in the lung epithelium overlying the fibrotic foci while LDHB levels were low in most IPF patient tissues. Collectively, our in vitro and IHC results consistently suggest that the alteration of LDHA/LDHB and GPR‐81 is associated with the fibrotic process and may contribute to the development and progression of lung fibrosis.

IPF is a disease of aging and it has been demonstrated that IPF fibroblasts exhibit a senescent phenotype. However, it is not clear how senescent IPF fibroblasts contribute to the maintenance and propagation of the persistent wound‐repair response that leads to progressive lung fibrosis. It has been postulated that senescent fibroblasts influence the phenotype of other cells via paracrine mechanisms in fibrosis and cancer; these effects have been attributed to the senescence‐associated secretory phenotype (Gonzalez‐Meljem et al., 2018; Waters et al., 2021). Although lactate has been traditionally regarded as a by‐product of the glycolytic pathway, recent studies suggest a potential direct pathophysiological role for lactate in the development of lung fibrosis (Kottmann et al., 2012; Newton et al., 2021). We found that control and IPF fibroblasts differ in both lactate production and lactate responsiveness under conditions of hypoxia. For example, under normoxic conditions, similar lactate levels were found in control and IPF fibroblasts. However, lactate expression was significantly increased in IPF fibroblasts when oxygen availability was reduced. Thus, it is a feasible concept that IPF fibroblasts behave as lactate donors and that the lactate secreted into the microenvironment by these aberrant cells can function as a signaling molecule to directly affect normal lung fibroblasts when oxygen is not readily available.

IPF fibroblasts are recognized to have substantial heterogeneity. A translational strength of our study is the use of multiple different primary control and IPF cell lines. Because these were de‐identified, we are not able to link results from individual cell lines with clinical parameters, although we note that all IPF fibroblast lines used in the study were from lung explants taken at the time of transplantation, so it is fair to presume that all patients had advanced lung fibrosis. We note that there is an ongoing knowledge gap in the field related to our understanding of how explant cell culture on rigid plastic and progressive passaging of cells impacts cell phenotype. Interestingly, however, topographical memory has been shown in fibroblasts cultured from distinct sites suggesting that there is some degree of phenotypic stability in these cells (Chang et al., 2002).

In addition to the potential effect of passaging primary cells, it is well recognized that culture conditions, including substrate stiffness and the extracellular matrix composition, can impact fibroblast phenotypes. Vitamin C is a key cofactor that regulates collagen synthesis and stability, and some studies suggest that Vitamin C can modulate TGF‐β signaling in fibroblasts (Piersma et al., 2017). We recognize that the impact of Vitamin C on matrix dynamics can have an effect on fibroblast phenotype over the course of experiments with longer durations. Although Vitamin C is present in the 10% FBS used in our cell culture conditions, in accordance with a robust literature on fibroblasts in the context of fibrosis and consistent with our prior studies on lung fibroblasts, we did not supplement media with additional Vitamin C (Desmouliere et al., 1993; Dodi et al., 2018; Lagares et al., 2017; Liu et al., 2015; Rahaman et al., 2014; Thannickal et al., 2003).

While the goal of our study was to evaluate the influence of hypoxia on fibroblast phenotype and our experiments were not designed to study the direct effects of hypoxia on TGF‐β signaling, a novel finding in our study was that TGF‐β treatment for 24 h did not have a significant effect on fibroblast differentiation under conditions of hypoxia. Canonical TGF‐β signaling is mediated by rapid phosphorylation of the receptor‐associated SMADs (SMAD2 and SMAD3, within 30 min) leading to phosphorylation of the common co‐SMAD, SMAD4, which traffics to the nucleus and serves as a transcription factor. Transcriptional responses to SMAD4 can be modulated by co‐activators and corepressors, including serum response factor (SRF) and myocardin‐related transcription factor‐A (MRTF‐A), both of which have been implicated in myofibroblast differentiation. We have shown that SMAD‐mediated activation of the non‐receptor tyrosine kinase, focal adhesion kinase (FAK), is essential for TGF‐β‐induced myofibroblast activation, that upregulation of α‐SMA is evident within 12 h of treatment, and that formation of organized alpha smooth muscle actin stress fibers, indicative of the myofibroblast phenotype, occurs within 24 h of treatment under the culture conditions used in this study (Hecker et al., 2011; Thannickal et al., 2003). As noted with Vitamin C, longer durations of culture following TGF‐β treatment can maintain and reinforce the myofibroblast phenotype through ongoing transcription‐mediated events as well as through TGF‐β‐independent signaling from the dynamic extracellular matrix. Importantly, while the myofibroblast was previously thought to be a terminally differentiated cell, there has been more recent appreciation for the plasticity of these cells with studies demonstrating that myofibroblasts can indeed de‐differentiate (Garrison et al., 2013; Hecker et al., 2011). We showed that under hypoxic conditions, control (but not IPF) fibroblasts have a reduction in α‐SMA expression. It remains to be determined whether this reduction represents myofibroblast de‐differentiation that does not occur in the IPF fibroblasts, or whether the normal fibroblasts under hypoxic conditions fail to differentiate.

Although our methods are consistent with those commonly used in studies of IPF fibroblasts, with current knowledge and technology, it is not yet feasible to sort primary lung fibroblasts to examine the different subpopulations in vitro. Thus, it would be informative to study the responses of different IPF fibroblast subpopulations under the hypoxic conditions in the future studies. TGF‐β impacts the expression of multiple glycolytic enzymes in fibroblasts and induces metabolic reprogramming characterized by aerobic glycolysis with increased extracellular acidification due to lactate generation under normoxic conditions in normal lung fibroblasts (Bernard et al., 2015; Xie et al., 2015). Emerging data indicate that while TGF‐β promotes pro‐fibrotic fibroblast phenotypes and that TGF‐β overexpression is sufficient for lung fibrogenesis, established fibrosis can be maintained and propagated by cell–matrix interactions and cell‐autonomous behaviors *independent* of persistent TGF‐β stimulation (Guzy et al., 2017). Moreover, accumulating studies show that IPF fibroblasts are transcriptionally, epigenetically, and phenotypically distinct from normal fibroblasts. We also found that TGF‐β maintained the ability to enhance extracellular lactate production under hypoxic conditions, indicating that hypoxia and TGF‐β can independently regulate fibroblast phenotype. Consistent with the differential and independent regulation of fibroblast phenotype by hypoxia and TGF‐β in normal and IPF fibroblasts, our studies indicate that hypoxia increases LDHA in normal and IPF fibroblasts while it suppresses LDHB only in the IPF fibroblasts. Under hypoxic conditions, TGF‐β did not have a significant effect on either LDHA or LDHB. Collectively, however, these findings suggest that hypoxia and TGF‐β can cooperatively enhance lactic acid production by fibroblasts through distinct mechanistic pathways.

Lactate has been implicated as an indirect contributor to lung fibrosis. For example, an acidic microenvironment can liberate latent TGF‐β from the extracellular matrix and contribute to fibrosis by increasing the pool of active TGF‐β (Kottmann et al., 2012). However, a recent study also reported that an acidic pH regulates Ogerin (GPR‐68)‐mediated inhibition of TGF‐β signaling (Bell et al., 2022), demonstrating the complexity of TGF‐β activation and signaling responsiveness. GPR‐68 is a G‐protein‐coupled receptor, and GPRs comprise the largest transmembrane receptor family in humans (Bockaert & Pin, 1999). As a class, GPRs are implicated in a variety of human diseases and targeting G‐protein‐coupled receptors and their downstream signaling has been proposed for the treatment of lung fibrosis, although GPRs can activate pro‐fibrotic or anti‐fibrotic signaling depending on the interaction of the associated G‐protein isoforms (Bell et al., 2022; Choi et al., 2021; Haak et al., 2020; Walker & Fisher, 2014; Zmajkovicova et al., 2020). Lactate is the ligand of GPR‐81 (HCAR1) (Kuei et al., 2011). Our data show that GPR‐81 expression is enhanced by exposure to hypoxia, and that GPR‐81 mediates lactate‐induced differentiation in normal, but not IPF, fibroblasts. These data suggest a novel paradigm in which lactate produced by IPF fibroblasts may function as a paracrine signal to promote myofibroblast differentiation in normal fibroblasts through ligation of GPR‐81.

We also found that lactate treatment maintained the ability to induce myofibroblast differentiation following adjustment of medium containing lactate to a neutral pH (pH 7.3), although the extent of α‐SMA induction was reduced compared to that from non‐pH adjusted medium. Thus, our findings suggest that lactate signaling through GPR‐81 functions as an additional mechanism, along with the potential effects of TGF‐β activation and direct regulation of pH sensors in the induction of myofibroblast differentiation. As lung tissues of patients with IPF have enhanced lactic acid concentrations, we suggest that each of these mechanisms may have a role in propagation of the myofibroblast phenotype and contribute to progressive pulmonary fibrosis in vivo.

Intercellular communication and cellular reprogramming are key underlying mechanisms of lung fibrogenesis. Such paracrine signaling for the induction of myofibroblasts may account for the geographically distinct development of fibroblast foci described in IPF (Jones et al., 2016) while also explaining how these discrete foci may integrate into an interconnected network, as has also been reported (Cool et al., 2006). Moreover, our studies suggest that the lactate receptor, GPR‐81, may represent a novel and specific target to halt the propagation of fibrosis. Although our novel study suggests that intercellular communication via lactate in response to the oxygen deficiency is a potential mechanism that propagates lung fibrosis, it is not clear how lactate decreases myofibroblast differentiation via GPR‐81 in control fibroblasts. Future studies will focus on the lactate shuttling and its mechanism that regulates myofibroblast differentiation at the molecular level. To summarize, we postulated that lactate produced by the alteration of a LDHA and LDHB in fibroblasts under hypoxic conditions could function as a critical signaling molecule. Our studies support this concept and demonstrate that normal and IPF fibroblasts respond differently under hypoxic conditions, and that under hypoxic conditions, lactate generated by IPF fibroblasts may directly affect the phenotype of neighboring normal fibroblasts. Finally, our studies identify a novel G‐protein‐coupled receptor (GPR‐81) that is induced by hypoxia, mediates differentiation of normal lung fibroblasts, and may present a novel target for intervention in fibrotic disease.

## AUTHOR CONTRIBUTIONS

Richard S. Nho drafted the article, designed, performed the experiments, analyzed the results, and wrote the article. Jeffrey C. Horowitz designed the experiments, analyzed the results, and wrote the article. Laszlo Farkas and Mauricio Rojas provided technical support. Camryn R. Rice, Jayendra Prasad, and Hannah Bone performed the experiments. All authors read and approved the current formation of article.

## FUNDING INFORMATION

This study was funded by the NIH/NHLBI HL114662 (RSN), NIH/NHLBI HL141195 (JCH), and 1 U01 HL14555‐01(MR).

## CONFLICT OF INTEREST STATEMENT

All authors declare no conflict of interest.

## ETHICAL APPROVAL AND CONSENT TO PARTICIPATE

This study was approved by the Ethics Committee of the Ohio State University. The use of human lung tissues was approved by the Institutional Review Board (IRB) at the Ohio State University (protocols 2017H0309 and 2021H0180) and all identifiers other than the diagnosis (IPF or “normal”) were removed from the specimens provided.

## SUPPLEMENTAL FILE CAN BE FOUND AT THE PRIVATE SHARING LINK

URL: https://figshare.com/s/8b30179f7f1b085292ef

DOI: https://doi.org/10.6084/m9.figshare.22189201

## Supporting information


Figure S1.

Figure S2.

Figure S3.

Figure S4.

Figure S5.

Figure S6.
