# Supplementary figures and images for: Persistent hypoxia promotes myofibroblast differentiation via GPR‐81 and differential regulation of LDH isoenzymes in normal and idiopathic pulmonary fibrosis fibroblasts
Source: Physiol Rep. 2023 Aug 31;11(17):e15759. doi: 10.14814/phy2.15759 (PMC10471601; doi:10.14814/phy2.15759)

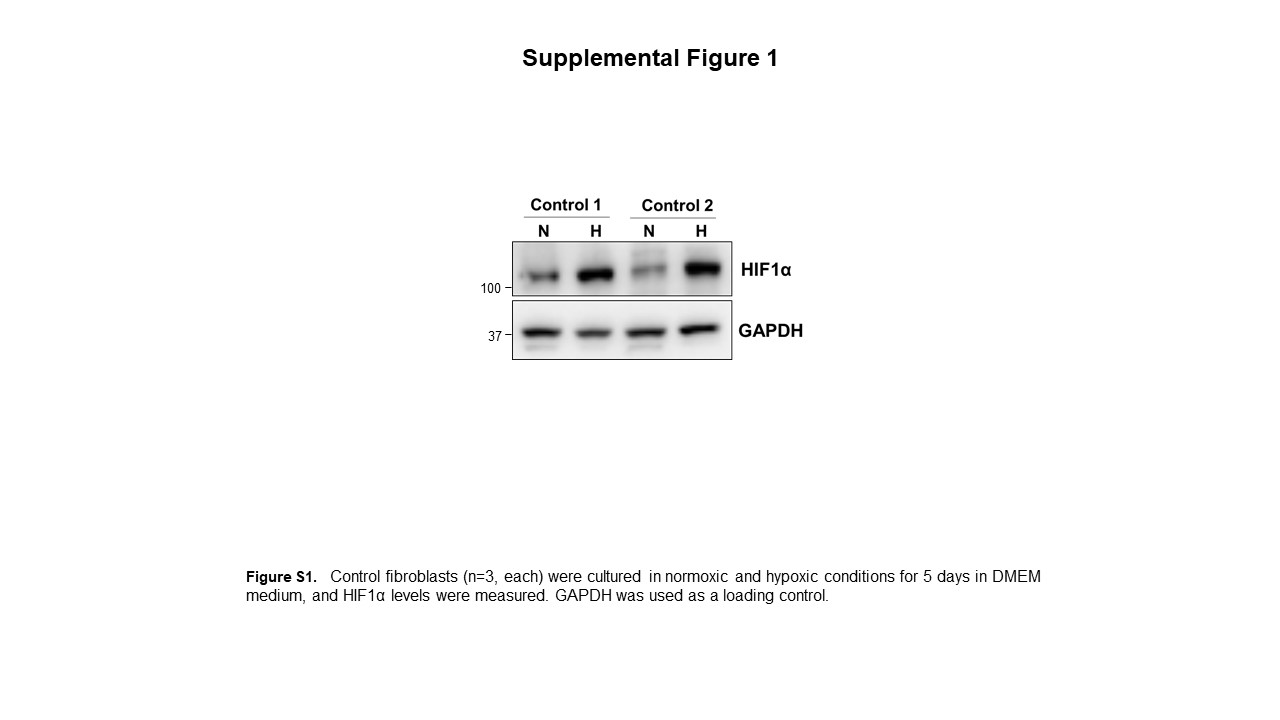

Supplement: Supplementary file 1 — Figure S1. [file PHY2-11-e15759-s003.jpg]

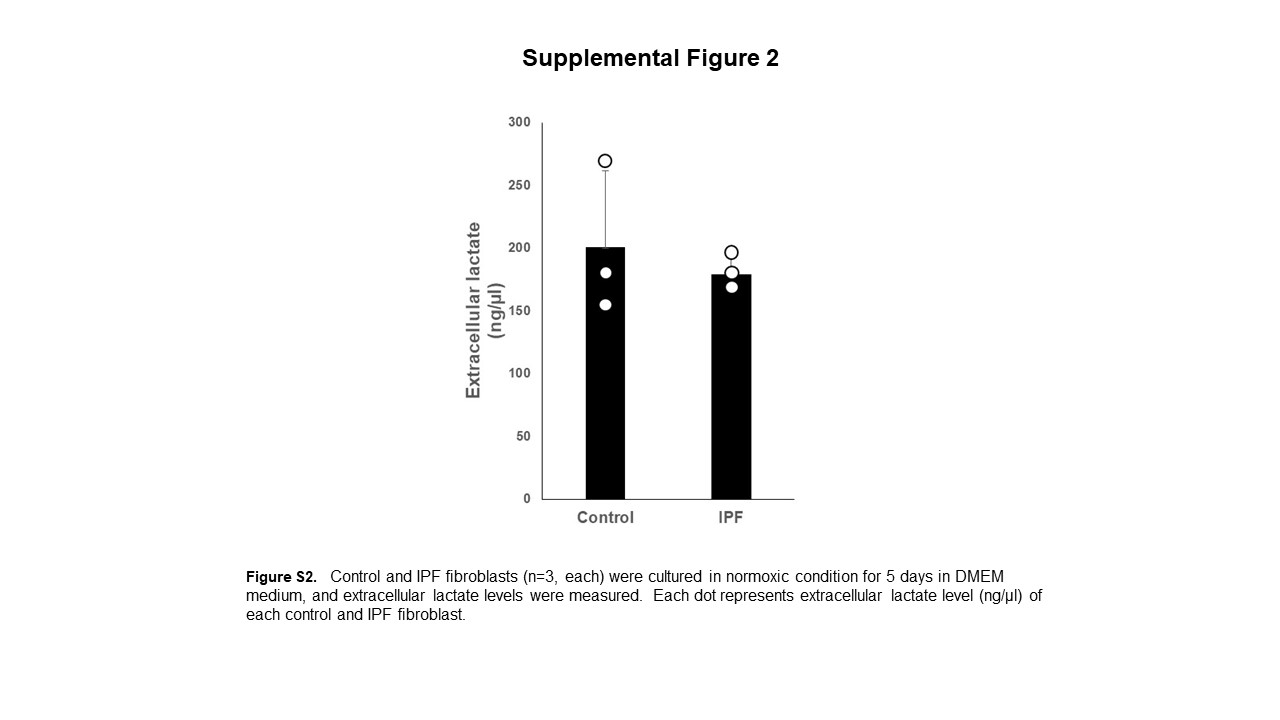

Supplement: Supplementary file 2 — Figure S2. [file PHY2-11-e15759-s001.jpg]

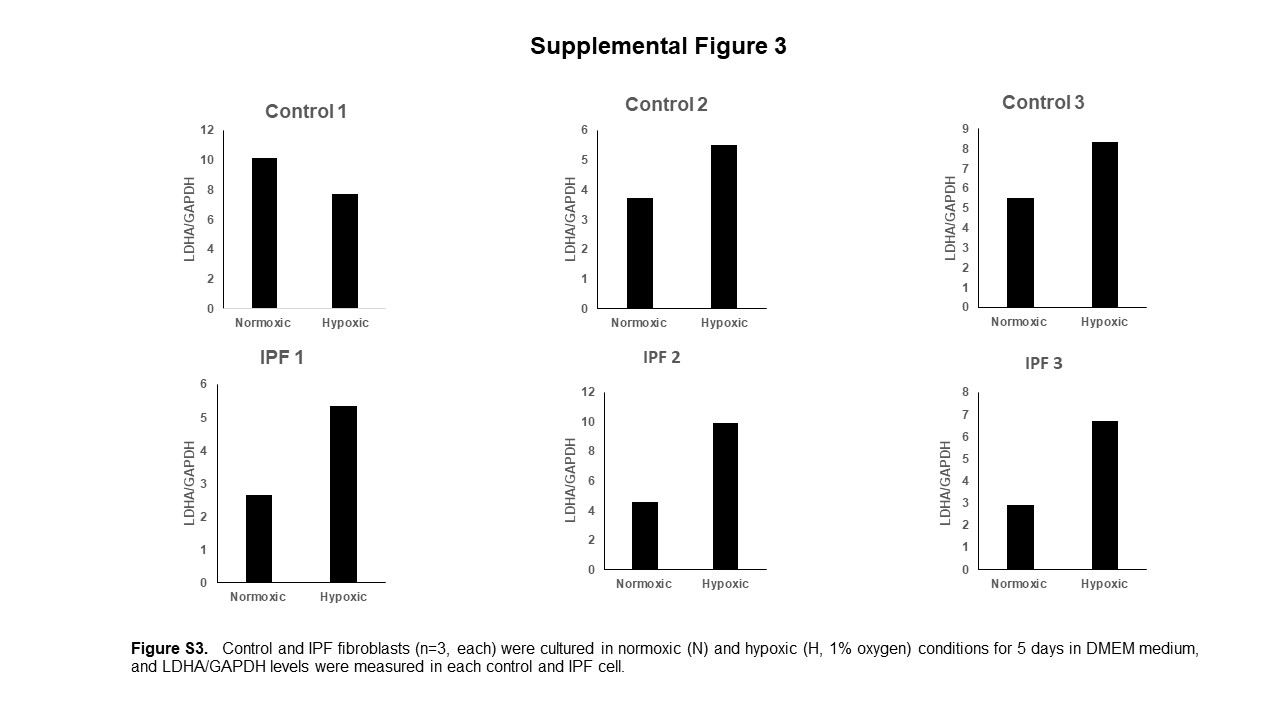

Supplement: Supplementary file 3 — Figure S3. [file PHY2-11-e15759-s005.jpg]

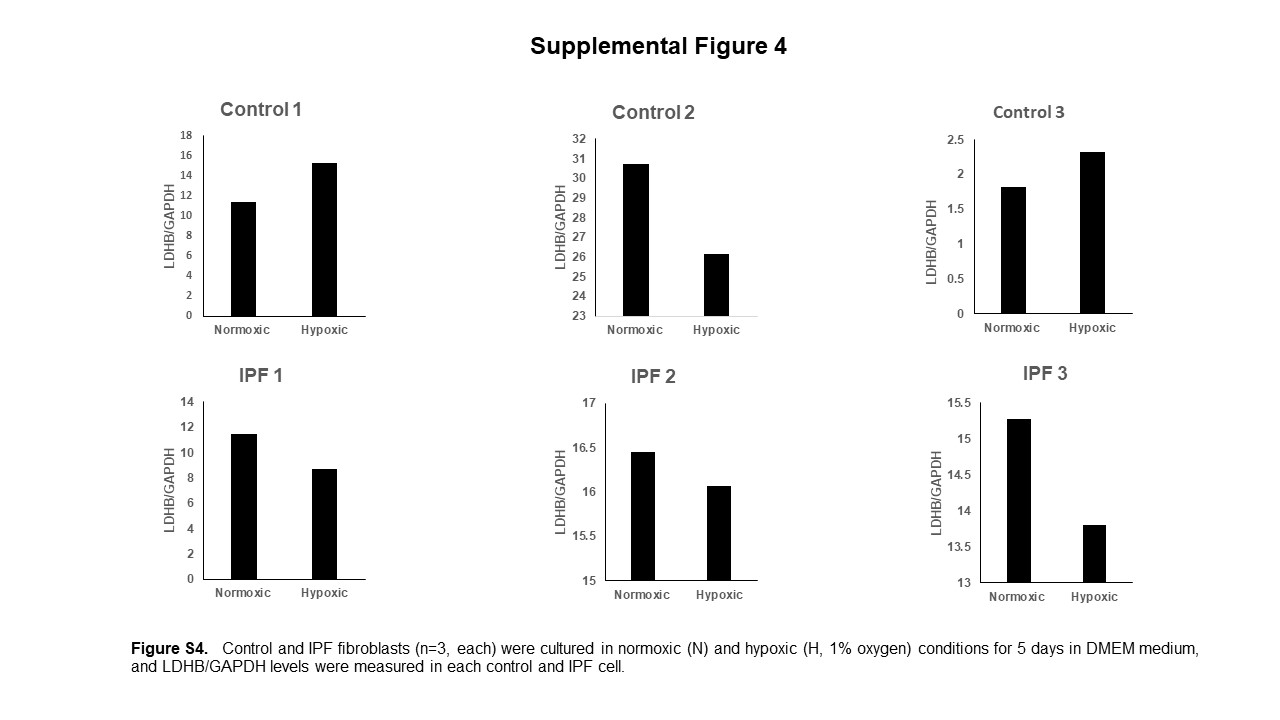

Supplement: Supplementary file 4 — Figure S4. [file PHY2-11-e15759-s006.jpg]

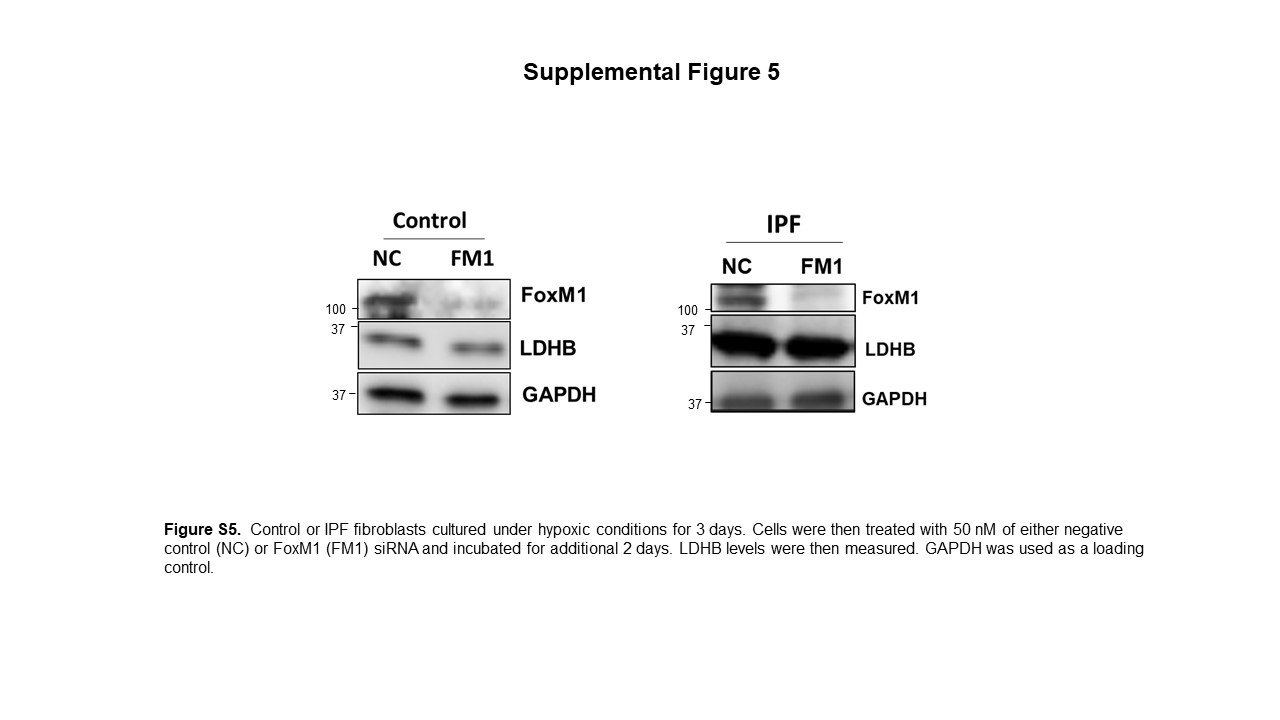

Supplement: Supplementary file 5 — Figure S5. [file PHY2-11-e15759-s002.jpg]

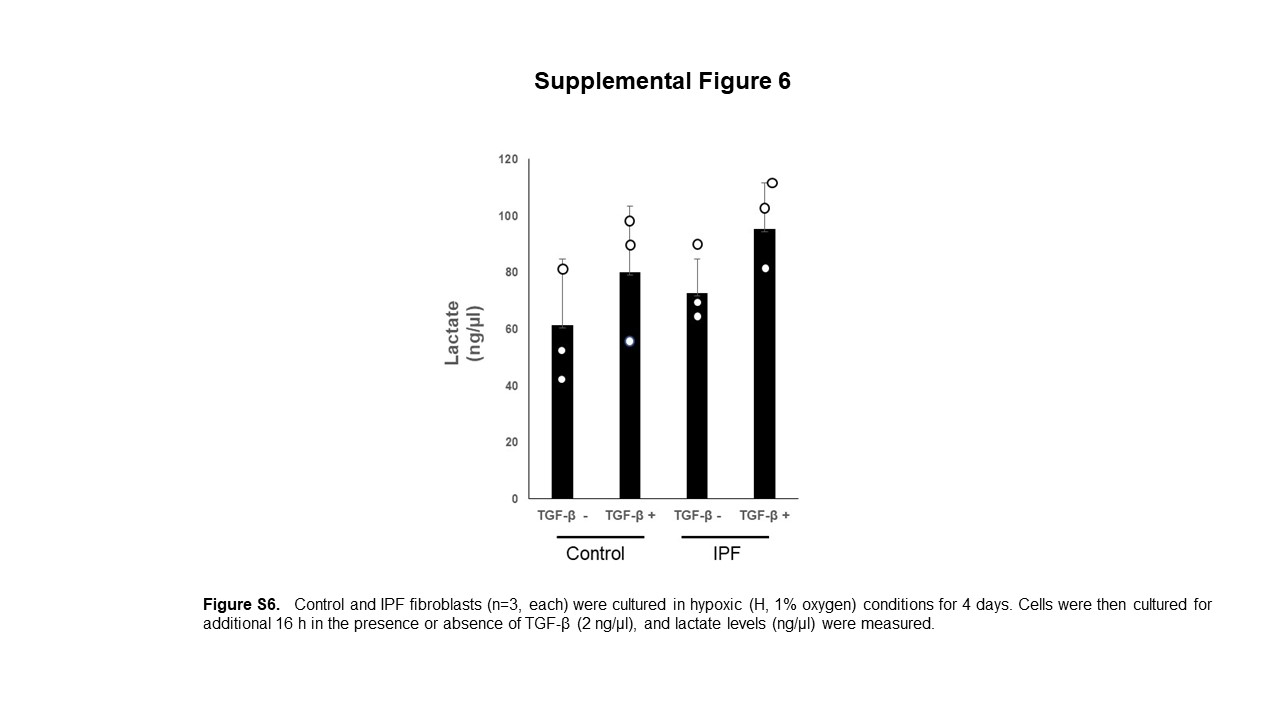

Supplement: Supplementary file 6 — Figure S6. [file PHY2-11-e15759-s004.jpg]
